# Supplementary material for: Integrated miRNA/mRNA Counter-Expression Analysis Highlights Oxidative Stress-Related Genes CCR7 and FOXO1 as Blood Markers of Coronary Arterial Disease
Source: Int J Mol Sci. 2020 Mar 12;21(6):1943. doi: 10.3390/ijms21061943 (PMC7139611; doi:10.3390/ijms21061943)
Supplement: Supplementary file 1 [file ijms-21-01943-s001.pdf]

## Supplementary

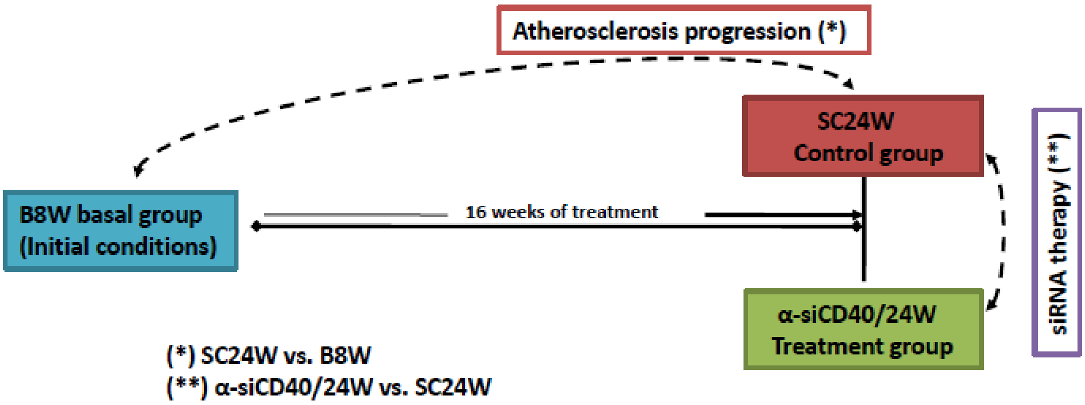


**Figure S1.** Experimental design to profile gene expression during Atherosclerosis progression and treatment in ApoE-null mice. Boxed are the three groups of animals used, initial (basal) and treated groups (control and treatment). Aortic tissue was extracted from mice of the different groups and used to prepare RNA to profile miRNA expression by TLDA and mRNA by microarray hybridization. Interrupted lines show the two experiments performed (group description in italics) to detect differentially expressed genes.

**Table S1.** MiRNAs and mRNAs whose expression was altered in the experiments of ATHp (SC24W vs. B8W) and treatment (α-siCD40/24W vs. SC24W). MiRNAs are identified by the name while mRNAs are only numbered. (*) MiRNAs whose expression was reversed by effect of the α-siCD40 treatment.

| **Atherosclerosis Progression (SC24W vs. B8W)** | |
|---|---|
| **Upregulated mi*RNAs* (1)** | **Downregulated mi*RNAs* (2)** |
| Mmu-let-7i-5p | Mmu-miR-30e-5p |
| Mmu-miR-10a-5p | |
| Mmu-miR-26a-5p (*) | |
| Mmu-miR-27a-3p | |
| Mmu-miR-27b-3p | |
| Mmu-miR-30a-5p (*) | |
| Mmu-miR-122-5p | |
| Mmu-miR-125b-5p (*) | |
| Mmu-miR-130a-3p | |
| Mmu-miR-324-5p | |
| Mmu-miR-465a-5p | |
| Mmu-miR-491-5p | |
| Mmu-miR-543-3p | |
| **Downregulated mRNAs** | **Upregulated mRNAs** |
| 1285 downregulated mRNAs | 1996 upregulated mRNAs |
| **TREATMENT (α-siCD40/24W vs SC24W)** | |
| **Upregulated miRNAs (4)** | **Downregulated miRNAs (3)** |
| Mmu-miR-582-3p | Mmu-miR-26a-5p (*) |

| | | |
|---|---|---|
| | Mmu-miR-30a-5p | (*) |
| | Mmu-miR-125b-5p | (*) |
| | Mmu-miR-363-3p | |

| **Downregulated mRNAs** | **Upregulated mRNAs** |
|---|---|
| 863 downregulated mRNAs | 780 upregulated mRNAs |

**Table S2.** GO enrichment analysis shows genes included in the top 20 functional categories recovered, most of them related to signaling pathways and to protein kinase binding and activity.

| **N** | **High level GO category** | **Genes** |
|---|---|---|
| 7 | Transferase activity | *MAP2K1 EGFR MAPK9 DAPK1 RNF7 MAPK1 MGAT3* |
| 6 | Drug binding | *DAPK1 MAP2K1 EGFR MAPK9 ATP2A2 MAPK1* |
| 6 | Small moleculebinding | *DAPK1 MAP2K1 EGFR MAPK9 ATP2A2 MAPK1* |
| 6 | Carbohydrate derivative binding | *DAPK1 MAP2K1 EGFR MAPK9 ATP2A2 MAPK1* |
| 3 | DNA-binding transcription factor activity | *GATA4 FOXO1 FOS* |
| 3 | Enzyme regulator activity | *MAP2K1 EGFR MAPK9* |
| 3 | Molecular function regulator | *MAP2K1 EGFR MAPK9* |
| 2 | MAP kinase kinase activity | *MAP2K1 MAPK1* |
| 2 | Transporter activity | *ATP2A2 KCNH3* |
| 2 | Transmembrane transporter activity | *ATP2A2 KCNH3* |
| 2 | Signaling receptor activity | *EGFR CCR7* |
| 2 | Molecular transducer activity | *EGFR CCR7* |
